# One planet: one health. A call to support the initiative on a global science–policy body on chemicals and waste

**DOI:** 10.1186/s12302-022-00602-6

**Published:** 2022-03-08

**Authors:** Werner Brack, Damia Barcelo Culleres, Alistair B. A. Boxall, Hélène Budzinski, Sara Castiglioni, Adrian Covaci, Valeria Dulio, Beate I. Escher, Peter Fantke, Faith Kandie, Despo Fatta-Kassinos, Félix J. Hernández, Klara Hilscherová, Juliane Hollender, Henner Hollert, Annika Jahnke, Barbara Kasprzyk-Hordern, Stuart J. Khan, Andreas Kortenkamp, Klaus Kümmerer, Brice Lalonde, Marja H. Lamoree, Yves Levi, Pablo Antonio Lara Martín, Cassiana C. Montagner, Christian Mougin, Titus Msagati, Jörg Oehlmann, Leo Posthuma, Malcolm Reid, Martin Reinhard, Susan D. Richardson, Pawel Rostkowski, Emma Schymanski, Flurina Schneider, Jaroslav Slobodnik, Yasuyuki Shibata, Shane Allen Snyder, Fernando Fabriz Sodré, Ivana Teodorovic, Kevin V. Thomas, Gisela A. Umbuzeiro, Pham Hung Viet, Karina Gin Yew-Hoong, Xiaowei Zhang, Ettore Zuccato

**Affiliations:** 1 UFZ Helmholtz Centre for Environmental Research, Permoserstraße 15, 04318 Leipzig, Germany; 2 Faculty Biological Sciences, Goethe University Frankfurt, Max-von-der-Laue-Straße 13, 60438 Frankfurt, Germany; 3 Catalan Institute of Water Research, Carrer Emili Grahit 101, 17003 Girona, Spain; 4 Spanish National Research Council, Institute for Environmental Assessment & Water Research, Water & Soil Quality Research Group, Jordi Girona 18-26, 08034 Barcelona, Spain; 5 Dept Environment & Geography, University of York, York, N Yorkshire, YO10 5DD UK; 6 Université de Bordeaux, 351 crs de la Libération, 33405 Talence, France; 7 Department of Environmental Sciences, Istituto di Ricerche Farmacologiche Mario Negri IRCCS, Via Mario Negri 2, 20156 Milan, Italy; 8 Toxicological Center, University of Antwerp, Universiteitsplen 1, 2610 Wilrijk, Belgium; 9 INERIS - Direction Milieu et Impacts sur le Vivant (MIV), Parc technologique ALATA, 60550 Verneuil-en-Halatte, France; 10 Center for Applied Geoscience, Eberhard Karls University of Tübingen, 72076 Tübingen, Germany; 11 Quantitative Sustainability Assessment, Department of Technology, Management and Economics, Technical University of Denmark, Produktionstorvet 424, 2800 Kgs. Lyngby, Denmark; 12 Department of Biological Sciences, Moi University, 3900-30100 Eldoret, Kenya; 13 Department of Civil and Environmental Engineering and Nireas-International Water Research Center, University of Cyprus, P.O. Box 20537, 1678 Nicosia, Cyprus; 14 Research Institute for Pesticides and Water, University Jaume I, 12006 Castellon, Spain; 15 RECETOX, Faculty of Science, Masaryk University, Kotlarska 2, Brno, Czech Republic; 16 Eawag, Swiss Federal Institute of Aquatic Science and Technology, 8600 Dübendorf, Switzerland; 17 Institute of Biogeochemistry and Pollutant Dynamics, ETH Zurich, 8092 Zurich, Switzerland; 18 RWTH Aachen University, Worringerweg 1, 52074 Aachen, Germany; 19 University of Bath, Bath, BA2 7AY UK; 20 School of Civil & Environmental Engineering, University of New South Wales, Sydney, NSW 2052 Australia; 21 Centre for Pollution Research and Policy, Department of Life Sciences, College of Health, Medicine and Life Sciences, Brunel University London, Uxbridge, UB8 3PH UK; 22 Institute for Sustainable Chemistry, Leuphana University Lüneburg, Universitätsallee 1, 21335 Lüneburg, Germany; 23 The French Water Academy, 51 rue Salvador-Allende, 92027 Nanterre, France; 24 Department Environment & Health, Vrije Universiteit Amsterdam, De Boelelaan 1085, 1081 HV Amsterdam, The Netherlands; 25 Departamento de Química Física, Facultad de Ciencias del Mar y Ambientales, Universidad de Cádiz – European Universities of the Seas, Campus Río San Pedro, 11510 Puerto Real, Cádiz Spain; 26 Institute of Chemistry, UNICAMP, Campinas, 13083-970 Brazil; 27 Université Paris-Saclay, INRAE, AgroParisTech, UMR ECOSYS, 78026 Versailles, France; 28 Institute for Nanotechnology and Water Sustainability (iNanoWS), College of Science, Engineering and Technology (CSET), University of South Africa, Pretoria, South Africa; 29 RIVM-National Institute for Public Health and the Environment, PO Box 1, 3720 BA Bilthoven, The Netherlands; 30 Department of Environmental Science, Radbound University Nijmegen, Nijmegen, The Netherlands; 31 Norwegian Institute for Water Research, Environmental Chemistry and Technology, Oslo, Norway; 32 Stanford University, Stanford, CA 94305-4020 USA; 33 Department of Chemistry & Biochemistry, University of South Carolina, Columbia, SC 29208 USA; 34 NILU-Norwegian Institute for Air Research, P.O. Box 100, 2027 Kjeller, Norway; 35 University of Luxembourg, 6 avenue du Swing, 4367 Belvaux, Luxembourg; 36 Institute for Social-Ecological Research (ISOE), Hamburger Alee 45, 60486 Frankfurt, Germany; 37 Environmental Institute, Okruzna 784/42, 97241 Kos, Slovak Republic; 38 Environmental Safety Center, Tokyo University of Science, 12-1 Ichigaya-Funagawara, Shinjuku, Tokyo 162-0826 Japan; 39 Nanyang Environment and Water Research Institute, Nanyang Technological University, Singapore, Singapore; 40 University of Brasilia, Brasília, DF 70910-000 Brazil; 41 Faculty of Sciences, University of Novi Sad, Novi Sad, Serbia; 42 Queensland Alliance for Environmental Health Sciences (QAEHS), The University of Queensland, 20 Cornwall Street, Woolloongabba, QLD 4102 Australia; 43 Faculty of Technology, UNICAMP, Limeira, 13484-332 Brazil; 44 VNU Key Laboratory of Analytical Technology for Environmental Quality, Vietnam National University, 334 Nguyen Trai, Hanoi, Vietnam; 45 Department of Civil and Environmental Engineering, National University of Singapore, 1 Engineering Drive 2, Singapore, Singapore; 46 Centre of Chemical Safety and Risks, School of the Environment, Nanjing University, Nanjing, China

**Keywords:** Chemical pollution, Science–policy body on chemicals, Planetary boundaries, One-health perspective, Systems thinking

## Abstract

The chemical pollution crisis severely threatens human and environmental health globally. To tackle this challenge the establishment of an overarching international science–policy body has recently been suggested. We strongly support this initiative based on the awareness that humanity has already likely left the safe operating space within planetary boundaries for novel entities including chemical pollution. Immediate action is essential and needs to be informed by sound scientific knowledge and data compiled and critically evaluated by an overarching science–policy interface body. Major challenges for such a body are (i) to foster global knowledge production on exposure, impacts and governance going beyond data-rich regions (e.g., Europe and North America), (ii) to cover the entirety of hazardous chemicals, mixtures and wastes, (iii) to follow a one-health perspective considering the risks posed by chemicals and waste on ecosystem and human health, and (iv) to strive for solution-oriented assessments based on systems thinking. Based on multiple evidence on urgent action on a global scale, we call scientists and practitioners to mobilize their scientific networks and to intensify science–policy interaction with national governments to support the negotiations on the establishment of an intergovernmental body based on scientific knowledge explaining the anticipated benefit for human and environmental health.

## A call to action

Climate change and biodiversity loss are well known to pose a threat to humankind and the global environment and are rightly in the focus of global policies and the public. However, a third major challenge on a global level of the same significance is the chemical pollution crisis that severely threatens human and environmental health globally and has not been sufficiently addressed by global and national policies. Governmental organization such as the European Commission [1, 2] and intergovernmental organizations such as the United Nations Environment Programme (UNEP) [3], have developed strategies and enacted legally binding regulations and multilateral agreements to control and manage chemical pollution to foster a toxic-free environment and enacted legally binding regulations, respective host the secretariats of legally binding multilateral agreements. Recently, UNEP published the first synthetic report, in which chemical pollution and wastes was listed as one of three top-priority issues together with climate change and biodiversity loss [4]. However, while international science–policy bodies are established to address climate change (Intergovernmental Panel on Climate Change, IPCC) and the loss of biodiversity (Intergovernmental Science–Policy Platform on Biodiversity and Ecosystem Services, IPBES), an overarching intergovernmental science–policy body to address pollution and its negative effects on humans and the environment on a global scale commensurate with the scope of the problem is still lacking.

Such a science–policy body on chemicals and waste has recently been suggested by several renowned environmental chemists and toxicologists, striving for enhanced bidirectional communication between policy-makers and scientists on a global scale with broad involvement of the wider scientific community to mobilize worldwide expertise to respond to this severe threat for humankind [5]. We strongly support this initiative. We highlight the need for horizon scanning and the establishment of early warning mechanisms on risks related to chemicals and waste to cover the growing universe of compounds and keep or reduce chemical pollution well below planetary boundaries for novel entities which include synthetic chemicals [6], but also to prevent exceedance of local and regional boundaries with clear impact on biodiversity, ecosystem services and human health. Immediate action to reduce global chemical pollution is essential and needs to be informed by sound scientific knowledge and data compiled and critically evaluated by an overarching science–policy interface body with wide involvement of scientists and practitioners as suggested by Wang et al. [5].

There is an increasing awareness that humanity, particularly the population and industry in high-income countries, have already likely left the safe operating space, i.e., transgressed the planetary boundary for novel entities [7]. In addition, international assessment and regulation of chemical pollution clearly lags behind the rapid and enormous increase in production and diversity of chemicals. Therefore, we see important tasks of the new body in improving prevention of pollution, reducing and eliminating data and management gaps on a global scale, identifying pollution problems with the potential to exceed regional and global boundaries, as well as developing strategies to tackle these issues holistically and systemically. Clearly communicating science and policy needs to solve this societal problem, the body is required to conduct assessments that go beyond current approaches, which are limited in terms of the geographical regions covered, the number of chemicals considered and the lack of considering ambient mixtures, the consideration of science-based and absolute pollution reduction targets and the lack of systems thinking. Major challenges for a novel science–policy body on chemicals and wastes are (i) to foster global knowledge production on exposure, impacts and governance, and go beyond data-rich regions (e.g., Europe and North America), (ii) to cover the entirety of hazardous chemicals and mixtures, (iii) to follow a one-health perspective considering the risks posed by chemicals on ecosystems, ecosystem services and human health, (iv) and to strive for solution-oriented assessments based on systems thinking and appreciating the complexity of driving forces, pressures, states, impacts and possible responses to reduce chemical pollution to remain within safe boundaries [7].

### Foster global knowledge on exposure and impacts

Several UN Sustainable Development Goals (SDGs) aim to globally ensure healthy lives (#3), access to clean water and sanitation (#6), responsible consumption and production (#12), and the protection of aquatic and terrestrial life (#14 and #15). Attaining these goals requires an efficient contaminant monitoring, control, and mitigation. Nine planetary boundaries have been identified including “novel entities” comprising new chemical substances, new forms of existing substances and modified and new life forms [8]. There is sufficient evidence for chemical impacts on environmental and human health on local to global scales [9], although its quantification is challenged by complexity [10, 11]. However, even if a well-defined planetary boundary for novel entities including chemical pollution is still lacking, the rate of increase of chemical production and use is alarming and exceeds that of most other indicators including population growth rate, emissions of carbon dioxide and agricultural land use [12]. A recent paper concluded that “humanity is currently operating outside the planetary boundary” on novel entities and that “the increasing rate of production and releases of larger volumes and higher numbers of novel entities with diverse risk potentials exceed societies’ ability to conduct safety related assessments and monitoring” [7]. At the global level, three criteria have been defined to be fulfilled to pose a threat to the Earth system [10]. Next to the (i) occurrence of a disruptive effect on a vital Earth-system process and (ii) a lack of reversibility, they include (iii) discovery only when the problem is already occurring at a global scale. One example for exceeding planetary boundaries may be plastic pollution combining global distribution and irreversibility [13] of the phenomenon with potential impacts on Earth systems [14, 15]. Extraordinary efforts are needed to mitigate plastic pollution and transform the global plastics economy [16] aiming at zero plastic pollution [17]. The excessive generation of plastic wastes generated worldwide (1.6 million tonnes per day) during the COVID-19 pandemic runs the risk to reverse the momentum of global efforts to reduce plastic waste production [18], resulting in severe pollution problems on all continents [19, 20]. Early warning strategies informed by monitoring data from many regions of the world, evaluated in assessments by the global scientific community, and organized in an international science–policy body is key to ensure or re-establish that the safe operating space for global societal development is not exceeded.

**Fig. 1:**
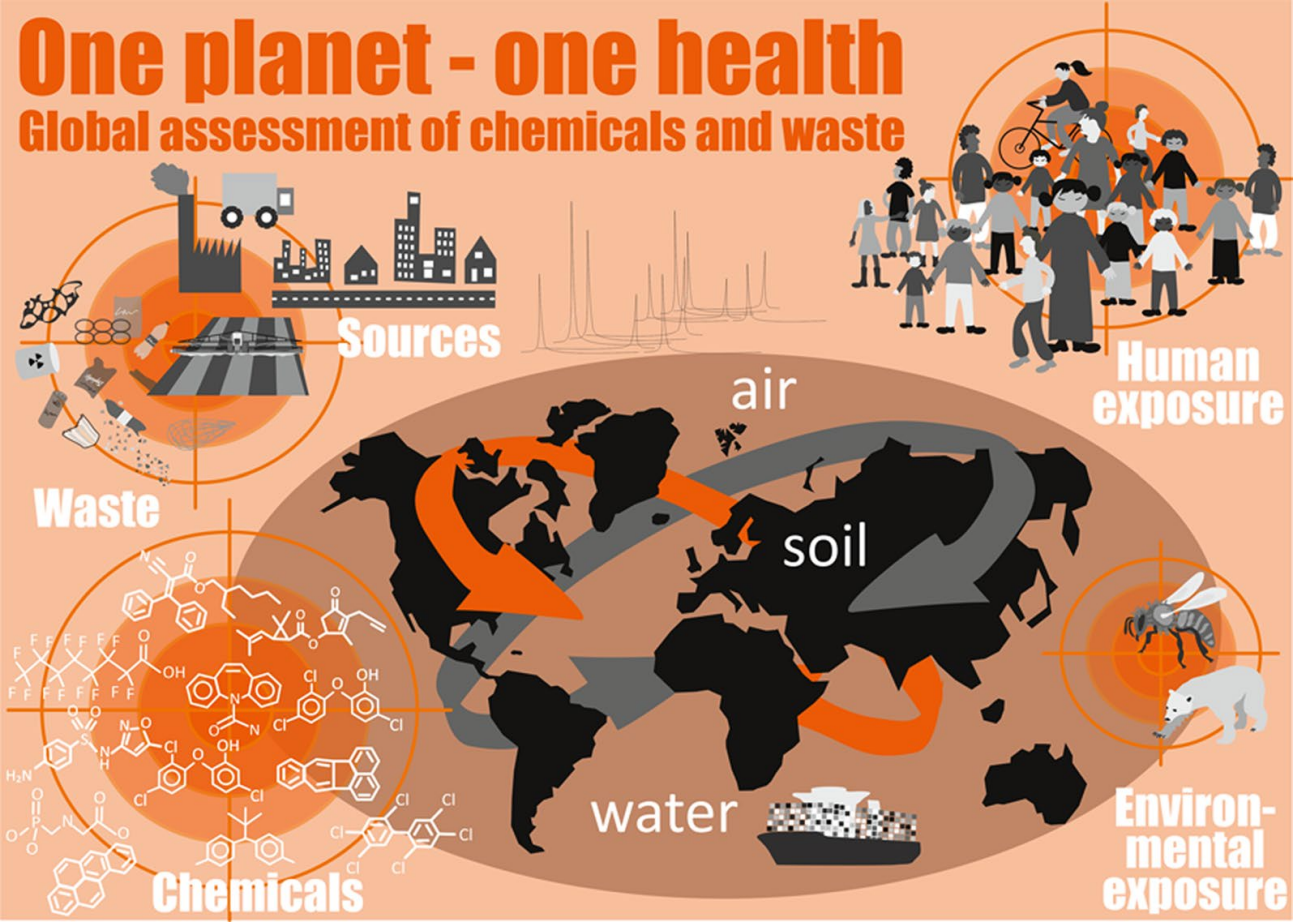
Global distribution of chemicals

Current separate approaches are insufficient. Existing data clearly support that chemical pollution and its impacts occur from the local to the global scale, despite current assessments and policies. Chemicals can be transported over long distances via the atmosphere and water cycles and hence affect regions far from where they were produced, used, or emitted (Fig. 1). Persistent organic pollutants have been detected in humans globally [21–24] and in their food [25], in aquatic biota even at the remotest places such as polar regions, high-mountain lakes, offshore waters and deep ocean trenches [26, 27] and in terrestrial food webs [28]. At the same time, there is evidence that climate change may remobilize legacy pollution in sediments [29] and glaciers [30] that has been thought to be permanently removed from the biosphere [31]. However, also less persistent chemicals of emerging concern (CECs), including pharmaceuticals and modern pesticides, occur ubiquitously in the global environment because of their widespread and continued use by societies all over the world [32–35].

The manufacture of hazardous chemicals is rapidly growing in low- and middle-income countries. Production is typically for use in high-income markets with poorly treated industrial wastewater discharged into domestic sewers [36]. Particularly high concentrations of hazardous chemicals are emitted from pesticide [37], textile [38] and drug [39] production. Manufacturing antibiotic drugs is often accompanied by very high concentrations in sewers that may act as a reservoir for antimicrobial resistant (AMR) bacteria [40]. Even if antimicrobials occurrence in the environment above Predicted No Effect Concentrations (PNEC) for resistance selection [41] remains a local phenomenon, the rapid spread of AMR bacteria by global mobility, migration and trade provides an almost perfect scenario for the exceedance of global boundaries [11]. It is predicted that by 2050, the number of deaths attributable annually to AMR bacteria will reach about 10 million, exceeding those of cancer, HIV and other diseases [42]. There is increasing evidence that even regional pollution problems can thus rapidly transform to global-scale issues that cannot be tackled at national and regional scales and require global action and steering globally by an international body.

While chemical pollution data in North America and Europe is increasingly becoming available, supported by continental scale science–policy networks such as the European NORMAN network [43], there is still a substantial lack of data from many countries in Asia, Africa and South America, as shown for pharmaceuticals [33] and pesticides [44], even if monitoring studies in data-poor countries such as Brazil [45], Sri Lanka [46], Kazakhstan [47], Nigeria [48] and Kenya [49] are slowly increasing. These emerging data indicate that concentrations of hazardous chemicals in low-income countries may be significantly higher than in Europe today. This is due to a combination of waste mismanagement [50] and global waste trade [51], poor sanitation and water treatment, the continued use and emission of high-risk chemicals phased out in high-income countries and the high use of region-specific compounds such as antiretroviral and antimalarial drugs and pesticides that may provide so-far unrecognized risks [52, 53].

Mitigating pollution problems in low-income countries is not only essential to protect human health, biodiversity, and ecosystem functions there, but has also direct benefits for all other regions. This effect may be highlighted for global trade of food, which has been shown to largely account for human exposure to pesticides and other hazardous chemicals in Europe and the US [54, 55]. Examples are the export of fruits and vegetables from South Africa and South America to Europe and transfer of meat from South America to Europe. The close nexus between unsustainable chemistry and agriculture for the production of food and other sectors for consumer goods with severe impacts on human health and ecosystems in producing regions, combined with the worldwide distribution of the hazardous chemicals with global trade, clearly demands for strategies on sustainable chemistry [56] on a global scale. An international body should carefully review existing regional strategies such as the EU Chemical Strategy for Sustainability [2]—including their regulatory mechanisms and effectiveness in mitigating pollution—and conclude on requirements for a toxic-free environment on a global scale. This overarching goal requires, among others, incentives and initiatives to close data gaps on pollution, risks and promising governance instruments in many regions of the world, supported amongst others by better uptake of digitalization methods [57] to derive and prioritize needs for global prevention, monitoring, regulation and mitigation.

## Cover the whole range of hazardous chemicals and mixtures

Since the 1970s, global production, trade and consumption of chemicals has increased substantially, particularly in emerging economies [12], and increasingly complex products have been designed to meet numerous functionalities [58]. A recent worldwide inventory revealed that more than 350,000 industrial chemicals and mixtures have been registered for production [59] and may finally end up in the environment. As most regulations handle per-chemical dossiers, restrictions for specific chemicals often result in their replacement by other, often equally persistent and hazardous chemicals, reflected by the emerging global distribution of these new compounds [60]. Although several international treaties including the Stockholm, Rotterdam, Minamata and International Maritime Organization (IMO) Conventions regulate the production, use and trade of persistent organic pollutants (POPs) and other hazardous substances, the large majority of potentially hazardous compounds in use [59] and detected in the environment [61] is not considered by any of these conventions.

Substantial progress in analytical multi-compound screening techniques opened new doors to extend monitoring to a large number of potentially hazardous target chemicals complemented by more exploratory non-targeted approaches and help to slowly approach the full complexity of the chemical pollution problem [34, 62]. At the same time, awareness is growing that chemicals exert impact on the local to the global scale as complex mixtures of a multitude of chemicals, and there is substantial evidence that ignoring mixture exposure and effects significantly underestimates pollution risks and impacts [63]. A better exchange on and understanding of the global ambient and human exposure to complex mixtures of chemicals is supported by new approaches of FAIR and open science [64, 65], openly accessible data infrastructures as provided by NORMAN [66, 67] and extensive web-based applications on chemical properties and hazard data for almost one million compounds such as the US-EPA CompTox Chemicals Dashboard [68] and PubChem [69]. These resources will allow for a quantum leap in the global data exchange, rapid growth of accessible knowledge and derivation of key management actions as required for effective assessments and the design of effective preventive and management actions by the suggested international science–policy body and for political decisions on pollution control and mitigation all over the world.

One of the great challenges for a novel science–policy body on chemical pollution and waste would be to respond to the rapidly increasing numbers of produced and used chemicals worldwide and develop strategies for a holistic approach on preventing, monitoring, regulating, and mitigating chemical pollution rather than chemical by chemical. Key elements of an unbiased strategy to explore pollution trends and upcoming risks may be the global promotion of non-target screening [62] and effect-based methods [34, 70] in environmental and human (bio)monitoring based on harmonized criteria in quality assurance [71]. These measures support grouping of chemicals for regulation and advanced assessment of chemical mixtures [72–74] and the restriction of potentially hazardous chemicals to essential use only [75].

## Follow a one-health perspective

Although the impact of chemical pollution on environmental and human health has historically been addressed separately, “the convergence of people, animals, and our environment has created a new dynamic in which the health of each group is inextricably interconnected” [76]. Environmental pollution is a key driver of human health impairment and at the same time of environmental health threats including losses of biodiversity and ecosystem functions and services to humans. Since humans and wildlife share many targets for biologically active chemicals [77] and adverse outcome pathways [78], problematic chemicals affect both, so that also innovative solutions for a pollution-free planet [3, 79] will protect both. Therefore, we suggest the new science–policy interface body to follow a one-health perspective addressing chemical risks on humans and ecosystems.

Diseases caused by chemical pollution have been estimated to be responsible for 9 million premature deaths in 2015, three times more than from HIV, tuberculosis and malaria together and 15 times more than from war and violence [80]. For neuro-developmental toxicity, a global pandemic has been uncovered with one in every six children having a neuro-developmental disability, including autism, attention deficit disorder, mental retardation, and cerebral palsy. Exposure to more than 200 neurotoxic chemicals has been identified as possible cause including metals, POPs and organic solvents [81]. Mixtures of polybrominated flame retardants have been shown to play an important role in neurodevelopmental effects [82]. Human reproduction is also at risk by chemical pollution. Within the last century a significant decline of total human fertility rates has occurred, while male reproductive disorders have increased [83, 84]. Exposure to mixtures of endocrine disruptors is hypothesized to be one of the drivers of this phenomenon [85].

Human health threats triggered by chemical pollution are typically accompanied by impairments of ecosystems and a decline of biodiversity [86, 87]. For Europe it has been shown that aquatic ecosystems are exposed to ambient mixtures of toxic pollution [88] at a level of which chemicals are of similar importance for the impaired ecological status as other well-accepted drivers, such as habitat degradation and excessive loads of nutrients [89]. In the oceans, legacy POPs still occur at concentrations that cause a continuous decline of distinct predatory marine mammals such as killer whales [90]. In freshwater ecosystems, continuously emitted endocrine disruptors may lead to population effects at very small concentrations, as demonstrated for contraceptive drugs which may cause intersex in wild fish [91] and collapse of fish populations [92]. Antifouling agents, globally used in high tonnages in ship paints [93], can act as endocrine disruptors and have been shown to cause the extinction of mollusc populations in harbours suffering from high exposure [94, 95]. In addition, they may also impair macrophyte communities [96] and even caused regime shifts in lake ecosystems [97].

The current biodiversity crisis has severe impacts on essential ecosystem services for humankind exceeding planetary boundaries for many biomes [98, 99]. This is particularly concerning for the drastic decline of flying insect biomass threatening pollination of the majority of plant species in nature and for food production, nutrient cycling and food sources for higher trophic levels [100]. Agriculture intensification, including increased pesticide and fertilizer usage, is one of the potential reasons for the decline of insects [100] and insectivorous grassland birds [101, 102]. The anti-inflammatory drug diclofenac applied in cattle was shown to cause near-extinction of vultures feeding on carcasses of animals treated with this compound in India and Pakistan [103], with severe effects on public health [104]. A strong link between ecosystem integrity and human health was also suggested for pesticide application in Africa. Pesticides has applied in Kenya have been shown not only to affect invertebrate communities but also to promote tolerant hosts for parasites and thus, pave the way for transmission of diseases such as schistosomiasis, with 218 million people infected worldwide and up to 280,000 deaths per year [105].

The close interlink between chemical pollution and impacts on human and environmental health, including losses of biodiversity and impaired ecosystem functions [106, 107], strongly demands for a one-health perspective from the local to the global scale. Thus, a global science–policy body on chemicals and waste should adopt this perspective from the very beginning and aim to maximize synergies of human and ecosystem health protection striving for a pollution-free and healthy planet [3, 79]. This goal requires involvement and collaboration of experts from the different scientific communities (chemistry, human health, (eco)toxicology, epidemiology, biodiversity, social sciences, economy) and the close collaboration with existing intergovernmental organizations such as the Strategic Approach to International Chemicals Management (SAICM), World Health Organization (WHO) and IPBES.

## Strive for solutions-oriented assessments based on systems thinking

Already established for pollution problems at the regional scale [108], the drivers–pressures–states–impact–response (DPSIR) causal–analytical scheme may be also useful to address this challenge at a global scale. Chemical emissions as a global pressure (P) for ecosystems and human health is highly complex with respect to the resulting mixture composition status (S), which may be dynamic in time and space but also regarding the associated potential impacts (I) on wildlife and human health. The diversity of driving forces (D) and actors involved in the emissions is large, and include agriculture, industry, global trade, and consumers, while those are in turn subjected to global change. Chemical pollution thus creates a high diversity of pollution states in different regions of the world with different impacts on biodiversity, ecosystem functions, exposure and health effects on human populations. It is then the focus on the response opportunities and consideration of a wide range of possible responses that matters for solving the problem, with potential solutions on all aspects of the DPSI-chain, i.e., on drivers, states and impacts. The earlier in that chain the response is effective, the less the risks and impacts.

We see the need for an international science–policy interface body on chemical pollution to take the high complexity of this system and the “solutions space” of possible responses into account from the very beginning [109]. Solution spaces can range from technical and management options for local application until governance options including regulatory and financing mechanisms at the global scale [110]. Systems thinking emphasizing the “how” and “why” of intervention outcomes should combine complexity-aware evaluation of monitoring data (critical mixture components, influence of time etc.) with broad stakeholder involvement and virtual simulation models that allow for scenario calculations [111]. Existing integrated fate-exposure models such as the UN Environment scientific consensus model USEtox may be used and expanded to test for different exposure and risk scenarios and possible interventions [112]. The power of these models to estimate near-field human exposures has been demonstrated recently by high-throughput screening of chemicals of concern in toys [113] and in building materials [114]. Long-range transport models for organic chemicals have been developed to understand pollution problems far from the regions, where chemicals have been produced and applied [115]. Consistent modelling frameworks for the distribution of chemical pollutants by global trade of goods and waste are less available although first examples exist such as the global food system [54].

## Our call to support the initiative on a global science–policy body

Along the lines discussed above, we see a clear need for the establishment of a global science–policy body on chemicals and waste, as suggested by Wang et al. [5], bringing together global scientific expertise on chemical pollution and governance, ecosystem and human health, as well as biodiversity to “strengthen the science–policy interface and the use of science in monitoring progress, priority-setting, solution focus and policy making throughout the life cycle of chemicals and waste” as suggested in the UNEP Global Chemicals Outlook II [79]. This is a call to scientists and practitioners to mobilize their scientific networks and to intensify science–policy interaction with national governments to support the negotiations on the establishment of an intergovernmental body based on scientific knowledge, explaining the urgency of global action on chemical pollution and discussing the anticipated benefit for human and environmental health on the way towards a pollution-free planet and a sustainable economic development within the safe operating space of the planetary boundaries. This initiative can only be successful if scientists and policy-makers join forces and combine expert and practical knowledge across continents and institutional silos in the suggested global panel to close the dramatic data gaps on chemical pollution in many parts of the world, identify the most important pollution problems and develop solution strategies to tackle them based on close science–policy interfacing and broad stakeholder involvement. A strong mandate and support from national governments and the international community are required to give prevention and mitigation of pollution an adequate weight in regulation, industry, and private behaviour to protect our common one health on our one planet.

## Data Availability

Not applicable.
